# Autophagy and Apoptosis Have a Role in the Survival or Death of Stallion Spermatozoa during Conservation in Refrigeration

**DOI:** 10.1371/journal.pone.0030688

**Published:** 2012-01-26

**Authors:** Juan M. Gallardo Bolaños, Álvaro Miró Morán, Carolina M. Balao da Silva, Antolín Morillo Rodríguez, María Plaza Dávila, Inés M. Aparicio, José A. Tapia, Cristina Ortega Ferrusola, Fernando J. Peña

**Affiliations:** 1 Laboratory of Equine Reproduction and Equine Spermatology, Veterinary Teaching Hospital, University of Extremadura Cáceres, Cáceres, Spain; 2 Department of Physiology, Faculty of Veterinary Medicine, University of Extremadura Cáceres, Cáceres, Spain; National Cancer Institute, United States of America

## Abstract

Apoptosis has been recognized as a cause of sperm death during cryopreservation and a cause of infertility in humans, however there is no data on its role in sperm death during conservation in refrigeration; autophagy has not been described to date in mature sperm. We investigated the role of apoptosis and autophagy during cooled storage of stallion spermatozoa. Samples from seven stallions were split; half of the ejaculate was processed by single layer centrifugation, while the other half was extended unprocessed, and stored at 5°C for five days. During the time of storage, sperm motility (CASA, daily) and membrane integrity (flow cytometry, daily) were evaluated. Apoptosis was evaluated on days 1, 3 and 5 (active caspase 3, increase in membrane permeability, phosphatidylserine translocation and mitochondrial membrane potential) using flow cytometry. Furthermore, LC3B processing was investigated by western blotting at the beginning and at the end of the period of storage. The decrease in sperm quality over the period of storage was to a large extent due to apoptosis; single layer centrifugation selected non-apoptotic spermatozoa, but there were no differences in sperm motility between selected and unselected sperm. A high percentage of spermatozoa showed active caspase 3 upon ejaculation, and during the period of storage there was an increase of apoptotic spermatozoa but no changes in the percentage of live sperm, revealed by the SYBR-14/PI assay, were observed. LC3B was differentially processed in sperm after single layer centrifugation compared with native sperm. In processed sperm more LC3B-II was present than in non-processed samples; furthermore, in non-processed sperm there was an increase in LC3B-II after five days of cooled storage. These results indicate that apoptosis plays a major role in the sperm death during storage in refrigeration and that autophagy plays a role in the survival of spermatozoa representing a new pro-survival mechanism in spermatozoa not previously described.

## Introduction

Artificial insemination is the reproductive technology most widely used in the equine industry since most of breed registries have accepted its use [1]. The objective of cooling stallion sperm is to extend the life expectancy of the spermatozoa, and thus be transported to breed mares located distant from the stallion. However cooled stallion spermatozoa last only for a few days viable and losses rapidly fertilizing ability [2]. Cooling imposes a number of stresses to the spermatozoa, collectively termed “cold shock” [3], [4] that finally leads to cell damage and/or death. This cold shock is attributed to lipid re-arrangements during cooling and again at warming. In addition during cooling storage, spermatozoa may suffer other insults such as lipid peroxidation [5], [6], [7], changes in pH [8], ATP depletion [9] and bacterial contamination [10], that after a variable time of storage finally ends in the death of most, if not all, spermatozoa. However the molecular mechanisms behind such sperm death remains largely unveiled. It is plausible to think that if the molecular mechanisms of sperm survival/death can be revealed [11], [12], improved strategies to store sperm during longer periods can be developed [13]. Autophagy is a conserved catabolic process primarily responsible for nonspecific degradation of redundant or faulty cell components. This occurs as part of cell's daily activities in response to metabolic or hypoxic stress and starvation, since to preserve normal cellular function a fast and responsive mechanism to degrade irreversibly damaged proteins is essential. Processed LC3B-II is a cellular readout of autophagy levels [14], [15]. LC3 is the commonly used name for microtubule associated protein 1 light chain. The unprocessed form of LC3 (pro LC3) is proteolytically cleaved by Atg4 protease, resulting in the LC3-I form with a carboxyterminal exposed glycine. Upon induction of autophagy the exposed glycine is conjugated by Atg7, Atg3 and by Atg12-Atg5 and Atg16L multimers to phosphatidylethanolamine (PE) moiety to generate LC3-II. To date LC3-II is the only well characterized protein that is specifically localized to autophagic structures throughout the process from phagophore to lysosomal degradation [15].

Death by apoptosis has been described in the stallion spermatozoa as a result of cryopreservation [16], [17], the same mechanism has been implicated in human [18] and bovine [19] sperm cryoinjury. However, there is no data regarding the molecular mechanisms leading to sperm death of stallion spermatozoa maintained in refrigeration. We hypothesize that apoptotic mechanisms may be also involved in the death of stallion spermatozoa during cooled storage. Furthermore we investigated and described for the first time the presence of autopaghy in stallion spermatozoa and propose a role of this mechanism in sperm survival.

## Materials and Methods

### Experimental design

The study evaluated sperm quality, apoptotic markers (motility, membrane integrity, membrane permeability, mitochondrial membrane potential, lipid peroxidation (LPO) phosphatydilserine translocation and caspase 3 and 7 activity) and autophagy in fresh semen maintained in refrigeration at 5°C up to five days. Motility and membrane integrity were evaluated daily, while the rest of sperm parameters were evaluated on days 1, 3 and 5 of storage. Autophagy was investigated on day 1 and on day 5. To have two distinct subpopulations with different sperm quality, two groups were established: extended semen (FE), and extended semen after colloidal centrifugation (CC); it has been demonstrated that the subpopulation of spermatozoa obtained after CC has better quality *in vitro*, survives longer and has better fertility [20], [21], [22]. All experiments were reviewed and approved by the Ethical committee of the University of Extremadura, Spain, ref AGL201020758, the only manipulation of animals was semen collection using standard procedures.

### Reagents and media

Ethidium homodimer, 5,5′,6,6′–tetrachloro-1,1′,3,3′ tetraethylbenzymidazolyl carbocianyne iodine (JC-1), YO-PRO-1, BODIPY 581/591C_11_, Caspase 3 and 7 detection kit and Alexa 488-Annexin-V were from Molecular Probes (Molecular Probes, Leiden The Netherlands), PureSperm Bottom® was from Nidacon, Sweden, Tris/Glycine/SDS buffer (10×) and Tris/Glycine buffer (10×) were from Bio-Rad (Richmond, CA). Complete, EDTA-free, protease inhibitor cocktail was from Roche Diagnostics (Penzberg, Germany). Antibody (Ab) anti-LC_3_B produced in rabbit (L7543) was purchased from Sigma-Aldrich (St. Louis, MO). Anti-rabbit monoclonal Abs: TNF-R1 (C25C1), TNF-R2 (3727) and TNF-alpha (D569) were purchased from Cell Signaling (Beverly, CA). Enhanced chemiluminescence detection reagents (SuperSignal West Dura), goat anti-rabbit IgG-HRP conjugated secondary antibody was from Pierce (Rockford, IL). Nitrocellulose membranes were from Schleicher & Schuell, BioSience (Keene, NH). Hyperfilm ECL was from Amersham (Arlington Heights, IL).

### Semen collection and processing

Semen (four ejaculates per stallion) was obtained from 7 Andalusian horses individually housed at the Veterinary Teaching Hospital of the University of Extremadura, Cáceres, Spain. Stallions were maintained according to institutional and European regulations, and were collected on a regular basis (two collections/week) during the 2011 breeding season. Ejaculates were collected using a Missouri model artificial vagina with an inline filter to eliminate the gel fraction, lubricated and warmed to 45–50°C. The semen was immediately transported to the laboratory for evaluation and processing. Half of the ejaculate was extended 1∶1 in INRA-96 centrifuged (600g×10 min) and re-suspended in INRA 96 to a final concentration of 50x10^6^ sperm/ml. The other half was processed by single layer colloidal centrifugation as previously described [21], [23] and extended in INRA-96 to 50x10^6^ sperm/ml.

### Sperm motility

Sperm kinematics was assessed using a CASA system (ISAS® Proiser Valencia Spain). The analysis was based on the examination of 25 consecutive, digitalized images obtained from a single field using×10 negative phase contrast objective and a warmed (37°C) stage. Semen was loaded in a 20 µm depth Leja chamber (Leja Amsterdam, The Netherlands). Images were taken with a time lapse of 1 sec - the image capture speed was therefore one every 40 msec. The number of objects incorrectly identified as spermatozoa were minimized on the monitor by using the playback function. With respect to the setting parameters for the program, spermatozoa with a VAP <15 µm/s were considered immotile, while spermatozoa with a velocity >15 µm/s were considered motile. Spermatozoa deviating <45% from a straight line were designated linearly motile and spermatozoa with a circular velocity (VCL) >45 µm/s were designated rapid sperm. Sperm motion absolute and re-calculated kinematic parameters measured by CASA included the following: Curvilinear Velocity (VCL) µm/s, Measures the sequential progression along the true trajectory. Linear Velocity (VSL) µm/s, Measures the straight trajectory of the spermatozoa per unit time. Mean Velocity (VAP) µm/s, Measures the mean trajectory of the spermatozoa per unit time. Average lateral head displacement (ALH) µm, Measures the mean head displacement along the curvilinear trajectory. BCF (Hz), *Number of times the sperm* head crosses the mean path/second.

### Staining for evaluation of sperm membrane integrity

The LIVE/DEAD® Sperm Viability Kit (Molecular Probes, Leiden The Netherlands) was used following the indications of the manufacturer. In brief, 1 to 5×10^6^ stallion spermatozoa were re-suspended in a final volume of 1 ml of HEPES buffered saline solution (10mM HEPES, 150mM NaCl, 10% BSA, pH 7.4). Sperm was then stained with 5 µL SYBR-14 (100nM) and incubated at 37° for 10 minutes in the dark and then samples were stained with 5 µL Propidium iodide (PI) (12 µM) and incubated for additional 10 minutes before reading in the flow cytometer. SYBR-14 fluorescence was detected in FL1 and PI fluorescence was detected in FL3.

### Assessment of subtle membrane changes and viability

The following stock solutions in DMSO were prepared: Yo-Pro-1, (25 µM), and Ethidium Homodimer-1 (1.167 mM) (Molecular Probes Europe, Leiden, The Netherlands), then one mL of a sperm suspension containing 5×10^6^ spermatozoa/mL was stained with 3 µL of Yo-Pro-1 and one µL of ethidium homodimer. After thorough mixing, the sperm suspension was incubated at 37°C in the dark for 16 min. This staining distinguishes four sperm subpopulations [17], [24], [25]. The first is the subpopulation of unstained spermatozoa. These spermatozoa are considered alive and without any membrane alteration. Another subpopulation is the Yo-Pro-1 positive cells emitting green fluorescence. It has been demonstrated that in early stages of apoptosis, there is a modification of membrane permeability that selectively allows entry of some non permeable DNA-binding molecules. This subpopulation are the spermatozoa showing early damage or a shift to another physiological state, since membranes become slightly permeable during the first steps of cryoinjury, enabling Yo-Pro-1 but not ethidium homodimer to penetrate the plasma membrane. None of these probes enters intact cells. Finally two subpopulations of cryoinjury-induced necrotic spermatozoa were easily detected, early necrotic, spermatozoa stained both with Yo-Pro-1 and ethidium homodimer (emitting both green and red fluorescence), and late necrotic spermatozoa, cells stained only with ethidium homodimer (emitting red fluorescence).

### Staining for detection of phosphatidylserine translocation Annexin-V assay

Phosphatidylserine (PS) translocation was detected with the use of Alexa Fluor 488 annexin V (Dead Cell Apoptosis Kit, Molecular Probes, Leiden The Netherlands), which detects the translocation of PS from the inner to the outer leaflet of the plasma membrane, and Propidium Iodide (PI) nucleic acids stain, a probe able to detect dead cells, and therefore exclude these cells from the analysis [26]. To 100 µL of semen, 500 µL of 5X annexin binding-buffer (4 parts of nanopure water and 1 part of commercial binding buffer: 50mM HEPES, 700mM NaCl, 12.5mM CaCl_2_, pH 7.4) was added. To this sample, 5 µL of Alexa Fluor 488 annexin V (solution in 25mM HEPES, 140mM NaCl, 1mM EDTA, pH 7.4, 0.1% BSA) and 10 µL of Propidium iodide (1mg/mL in deionized water) were added. After 15 minutes of incubation in the dark at room temperature, 400 µL of 5X annexin binding-buffer were added. Cytometry analysis was then performed. Apoptotic cells show green fluorescence (530nm), dead cells show red fluorescence (620nm) and live cells have no fluorescence, after excited with Argon-laser ion of 488nm.

### Staining for detection of active caspases 3 and 7

The caspase FITC-DEVD-FMK (caspase 3 and 7) *in situ* marker (Molecular Probes, Leiden, The Netherlands) was used to detect active caspases. This cell-permeable specific caspase inhibitor peptide is conjugated to fluorescein isothiocyanate (FITC) and binds covalently to active caspases 3 and 7 serving as *in situ* marker for apoptosis [17], [27]. A sample of 5x10^6^ spermatozoa were suspended in 1mL of PBS, and -after adding one µL of FITC DEVD-FMK (5mM) the suspension was incubated at room temperature (22–25°C) in the dark for 20 minutes. After incubation, the spermatozoa were washed with PBS, followed by the addition of 1 µL of ethidium homodimer (1.167 mM) (Molecular Probes Europe, Leiden, The Netherlands) to detect membrane damage. Flow cytometry was conducted within 10 minutes.

### Staining for detection of lipid peroxidation

Lipid peroxidation was measured using the probe BODIPY 581/591 C_11_ (Molecular Probes) as previously described in our laboratory [17], [28]. BODIPY 581/591 C_11_ is a fatty acid that is a sensitive fluorescent reporter for LPO, undergoing a shift from red to green fluorescence when in presence of this process, due to the oxidation of the phenylbutadiene segment of the fluorophore. This permits fluorescent ratio emission of live cells. A suspension of 2x10^6^ spermatozoa in PBS was loaded with the probe at a final concentration of 2 µM. Spermatozoa were then incubated at 37°C for 30 minutes, washed by centrifugation at 1000 rpm during 5 minutes to remove the unbound probe, and analyzed using a flow cytometer. Positive controls were obtained after the addition of 80 µM ferrous sulfate to additional sperm suspensions.

### Evaluation of mitochondrial membrane potential (ΔΨm)

The lipophilic cationic compound 5,5′,6,6′–tetrachloro-1,1′,3,3′ tetraethylbenzymidazolyl carbocianyne iodine (JC-1) has the unique ability to differentially label mitochondria with low and high membrane potential [29], [30]. In mitochondria with high membrane potential, JC-1 forms multimeric aggregates emitting in the high orange wavelength of 590 nm, when excited at 488 nm. In mitochondria with low membrane potential, JC-1 forms monomers, these monomers emit in the green wavelength (525 to 530 nm) when excited at 488 nm. For staining a 3mM stock solution of JC-1 (Molecular Probes Europe, Leiden, The Netherlands) in dimetilsulfoxide (DMSO) was prepared. From each sperm sample, 1 mL of a sperm solution in PBS containing 5×10^6^ cells/ mL was stained with 0.5 µL JC-1 stock solution. The samples incubated at 38°C in the dark for 40 minutes before flow cytometric analysis.

### Flow Cytometry

Flow cytometric analyses were carried out with a Coulter EPICS XL (Coulter Corporation Inc., Miami, FL, USA) flow cytometer equipped with standard optics. The standard four color set up uses 525, 575, 620 and 675 nm band pass filters and 488, 550, 600 and 645nm dichroic long pass filters. The system has an argon-ion laser (Cyonics, Coherent, Santa Clara, CA, USA) performing 15 mW at 488 nm and EXPO 2000 software. Subpopulations were divided by quadrants, and the frequency of each subpopulation was quantified. Forward and sideways light scatter were recorded for a total of 10,000 events per sample. Non sperm events were calculated and eliminated as previously described [31]. Samples were measured at a flow rate of 200–300 cells/sec. Green fluorescence was detected in FL1(525 nm band pass filter) red fluorescence was detected in FL3 (620 nm band pass filter), and orange fluorescence in FL2 (575 nm band pass filter).

### Western blotting

During autophagy, LC3B-I is processed and converted to LC3B-II, thus the amount of LC3B-II is correlated with the number of autophagosomes [14].Western blotting was performed as previously described [32], [33]. Briefly, stallion semen was centrifuged and washed twice with PBS. After washing, sperm cells were sonicated for 5 sec at 4°C in 100 µl of Lysis Buffer consisting in 50 mM Tris/HCl, pH 7.5, 150 mM NaCl, 1% Triton X-100, 1% deoxycholate, 1 mM EGTA, 0.4 mM EDTA, a protease inhibitor cocktail (Complete, EDTA-free), and 0.2 mM Na_3_VO_4_. The homogenates were clarified by centrifugation at 10,000×g (15 min, 4°C) and the supernatant was used for analysis of protein concentration followed by dilution with 4× SDS sample buffer. Proteins (25 µg/well) from stallion sperm lysates were fractionated by SDS-PAGE using 4-20% polyacrylamide gradient gels and transferred to nitrocellulose membranes. After blocking, membranes were incubated overnight at 4°C with anti-LC_3_B (1∶2000). The following day, membranes were washed twice and incubated for 45 min at 25°C with anti-rabbit IgG -HRP conjugated secondary Ab. Membranes were then washed again, incubated with enhanced chemiluminescence detection reagents, and, finally, exposed to Hyperfilm ECL films (Amersham). The intensity and molecular weight of appearing bands were quantified using the software Scion Image for Windows, version 4.02 (Scion Corp., Frederick, MD), normalized to β actin values.

### Immunocytochemistry

Spermatozoa were washed and suspended in PBS adjusting the cell concentration to 1x10^6^ cells per ml. Fifteen µl of the sperm suspension were spread on poly-l-lysine coated slides and allowed to attach for 10 min. Cells were then fixed with 3% formaldehyde in PBS for 15 min at room temperature and permeabilized with 0.2% Triton X-100 in PBS for 5 min. Slides were washed three times for 10 min with PBS and incubated in PBS supplemented with 5% BSA (w/v) for 90 min to block non-specific sites. After blocking, slides were incubated overnight at 4°C with anti-TNF-R1 (1∶100), anti-TNF-R2 (1∶100) or anti-TNF-alpha (1∶100) antibodies diluted in PBS containing 5% BSA (w/v). Next day samples were extensively washed with PBS and further incubated with a goat anti-rabbit Alexa 488-conjugated antibody for 45 min at room temperature. Finally, slides were washed with PBS and examined with a Bio-Rad MRC1024 confocal microscope with a X60 objective in oil immersion. Samples were excited at 488 nm with an argon laser and emission was recorded using a 515-nm longpass filter set. Processing the samples without primary antibody assessed the absence of non-specific staining.

### Statistical analysis

Data were first examined using the Kolmogorov-Smirnov test to determine their distribution, a multivariate analysis of variance was performed (ANOVA) and when significant differences were found, the non-parametric Mann-Whitney U-test was used to compare pairs of values directly if data did not adjust to a normal distribution. All analyses were performed using SPSS version 17.0 for Windows (SPSS Inc., Chicago, IL). The Spearman non-parametric test was used to study the correlations among apoptosis and autophagy and the results of the sperm analysis. Significance was set at P<0.05.

## Results

### Sperm motility and kinematics

As expected all parameters of sperm motility and kinematics decreased over the incubation period (Table 1). The percentages of total motile sperm and progressive sperm decreased in the second day of incubation (p<0.01), however on day 3 the percentage of total motile sperm was not significantly different from day 2. The percentage of progressive motile sperm also decreased the second and third day of incubation (p<0.01), but the difference between the forth and fifth day was not statistically significant. Sperm velocities and the percentage of rapid sperm also decreased over the incubation period. VCL significantly decreased on the second day (p<0.01), but VCL on the second day was not different from VCL on the third day. On the other hand, VLS and VAP also decreased on the third day of storage (p<0.01). ALH and BCF only changed significantly (p<0.01) at the end of the period of storage; in addition ALH was significantly influenced by colloidal centrifugation that significantly reduced this parameter (p<0.01).

**Table 1:** Sperm motility and kinematics after computer assisted sperm analysis (CASA) of stallion spermatozoa stored during five days (day 1 D1 to day 5 D5) at 5°C FE fresh extended sperm, CC sperm processed through colloidal centrifugation.

	D1		D2		D3		D4		D5	
	FE	CC	FE	CC	FE	CC	FE	CC	FE	CC
TM%	84.2±11.1^a^	88.7±8.5^ a^	65.4±14.1^e^	72.9±18.6^ e^	60.9±16.5^b,e^	57.8±23.5^b,e^	48.6±17.3^ b^	47.1±26.9^ b^	41.7±20.6^c^	37.4±21.2^c^
LM%	55.7±11.9^a^	66.0±12.5^a^	38.1±15.9^e^	49.2±22.5^ e^	29.9±11.3^c^	29.1±21.5^c^	20.1±11.9^d^	19.8±16.2^d^	14.4±7.3^d^	10.1±9.9^d^
RS%	34.9±15.1^a^	44.9±19.7^a^	14.2±8.7^e^	22.7±15.0^e^	10.9±8.0^ed^	13.5±14.8^ed^	6.5±4.06^d^	9.1±8.6^d^	6.7±5.8^d^	4.7±5.5^d^
VCLµm/s	83.2±18.4^a^	90.8±19.5^a^	62.3±15.2^e^	68.8±22.1^e^	58.4±15.0^ed^	57.2±17.4^ed^	53.7±13.4^d^	50.9±19.2^d^	53.5±14.1^d^	44.5±20.1^d^
VSLµm/s	34.1±9.0^a^	44.9±11.4^a^	25.5±10.2^e^	32.8±12.5^e^	21.2±6.9^c^	22.1±10.4^c^	18.1±5.7^cd^	17.5±8.7^cd^	15.0±3.9^d^	14.1±8.1^d^
VAPµm/s	48.9±14.4^a^	60.9±17.0^a^	34.2±11.4^e^	41.9±14.5^ e^	29.7±8.1^c^	30.1±11.9^c^	26.3±6.1^c^	25.8±9.6^c^	24.0±5.6^c^	21.7±9.6^c^
ALHµm	3.7±0.6^a^	3.6±0.8^a^	3.4±0.5^a^	3.2±0.5^a^	3.5±0.4^a^	3.4±0.4^a^	3.7±0.3^a^	3.4±0.8^b^	4.3±0.4^b^	3.3±1.31^a^
BCF Hz	11.1±2.1^a^	10.7±1.7^a^	10.4±2.1^a^	11.5±1.7^a^	10.3±2.1^a^	11.1±1.9^a^	9.6±2.1^ab^	9.4±3.7^ab^	8.4±1.1^b^	8.7 ±3.7^b^

TM% total motile sperm, LM% linear motile sperm, RS% rapid sperm, VCL circular velocity, VSL straight line velocity, VAP average velocity, ALH lateral head displacement, BCF beat cross frequency. Within a row values with different superscripts differ statistically a-e, P<0.01. (means ± SD) Results are derived from 28 identical experiments (7 stallions, 4 ejaculates per stallion).

### Sperm membrane integrity

There was not a significant decrease in the percentage of live sperm over the period of cooled storage. The percentage of live sperm at the end of the storage period was above 70% both in fresh extended sperm and in sperm subjected to CC. The overall percentage of live sperm was higher in CC selected sperm (p<0.01) (table 2).

**Table 2:** Sperm membrane integrity (SYBR-14/PI) of stallion spermatozoa stored during five days (day 1 D1 to day 5 D5) at 5°C FE fresh extended sperm, CC sperm processed through colloidal centrifugation.

	D1		D2		D3		D4		D5	
	FE	CC	FE	CC	FE	CC	FE	CC	FE	CC
LIVE %	78.7±13.1^a^	84.7±11.3^b^	82.9±7.7^a^	88.8±6.7^b^	73.8±13.1^a^	81.6±5.3^b^	74.0±9.17^a^	80.3±11.0^b^	72.1±7.7^a^	74.4±14.0^a^
DEAD%	16.3±10.3	10.0± 8.1	12.5±7.3	6.6±7.0	17.9±11.9	11.5±6.1	17.5±7.2	12.2±9.5	19.0±8.8	17.9±13.1
DAMAGED%	5.0±5.9	5.2±9.5	4.5±2.8	3.9±5.6	8.2±7.7	6.9±6.7	8.5±8.9	7.4±8.9	8.8± 7.7	7.6±6.5

LIVE % (SYBR-14+ sperm), DEAD% (PI+ sperm), DAMAGED, (SYBR-14+/PI+sperm). Within a row values with different superscript differ statistically a-b p<0.01. (means ± SD) Results are derived from 28 identical experiments (7 stallions, 4 ejaculates per stallion).

### Membrane intactness and subtle changes in membrane permeability

Colloidal centrifugation increased the percentage of spermatozoa showing intact membranes (p<0.01). The percentage of intact membranes decreased on day 3 of storage, but only in the CC group, while in the FE group the percentage on intact spermatozoa decreased after 5 days of storage (table 3). The change in the percentage of intact spermatozoa was parallel to change in the percentage of apoptotic spermatozoa that significantly increased in both groups at the end of the period of storage (p<0.01).

**Table 3:** Membrane intactness and subtle changes in sperm membrane integrity of stallion spermatozoa stored during five days (day 1 D1 to day 5 D5) at 5°C FE fresh extended sperm, CC sperm processed through colloidal centrifugation.

	D1		D3		D5	
	FE	CC	FE	CC	FE	CC
Intact%	58.1±12.9^c^	72.4±13.2^a^	51.3±9.9^c^	60.4±9.1^c^	45.8±11.7^b^	49.9±11.0^b^
YoPro+ %	12.7±6.7^a^	8.6±2.9^a^	9.4±2.7^a^	8.41±3.3^a^	20.7±18.9^b^	18.5±10.8^b^
YoPro+/Eth+ %	21.6±12.9	12.3±11.1	28.2±9.9	18.5±3.7	22.9±13.1	25.9±9.9
Eth+ %	7.5±6.2	6.6±7.0	11.0±8.6	12.8±6.2	10.5±10.3	5.5±4.6

Intact spermartozoa are those not stained and thus represent spermatozoa with completely intact membranes. YoPro+ are early apoptotic sperm depicting an increase in sperm membrane permeability, YoPro+/Eth+ are late apoptotic and Eth+ are necrotic spermatozoa. Within a row values with different superscript differ statistically a-c p<0.01. (means ± SD) Results are derived from 28 identical experiments (7 stallions, 4 ejaculates per stallion).

### Active caspases 3 and 7

High caspase 3 and 7 activity was found in stallion spermatozoa, ranging from 19 to 30%. There was no effect of CC or days of storage in the percentage of spermatozoa showing high caspase 3 and 7 activity (table 4).

**Table 4:** Active caspases 3 and 7 of stallion spermatozoa stored during five days (day 1 D1 to day 5 D5) at 5°C FE fresh extended sperm, CC sperm processed through colloidal centrifugation.

	D1		D3		D5	
	FE	CC	FE	CC	FE	CC
High activity%	27.9±31.8	28.3±32.6	18.9±15.9	18.2±25.3	30.2±28.9	28.3±22.9
Low activity%	43.7±27.7	48.6±35.3	37.9±15.0	42.61±9.7	34.6±30.7	34.6±24.4
Dead sperm%	28.6±17.7^a^	23.3±19.7^a^	43.5±4.5^b^	40.8±8.8^b^	35.4±18.8^ab^	37.5±10.6^ab^

High activity are spermatozoa with high caspase 3 an 7 activity, low activity are spermatozoa with low caspase 3 and 7 activity and dead sperm correspond to necrotic (Ethidium positive spermatozoa). Within a row values with different superscript differ statistically, a-b p<0.01. (means ± SD) Results are derived from 28 identical experiments (7 stallions, 4 ejaculates per stallion).

### Detection of phosphatidylserine (PS) translocation/Annexin assay

The percentage of intact sperm (A-/PI-) decreased at day 3 of storage (p<0.01), but did not decrease further on day 5. The percentage of necrotic sperm (A-/PI+) followed the opposite trend, increasing on day 3 but no further on day 5 (table 5). The percentage of annexin+ sperm did not vary over the time of storage while the percentage of A+/PI+spermatozoa increased on the 5^th^ day (p<0.01). CC had no effect on the percentage of spermatozoa showing PS translocation.

**Table 5:** Annexin-V assay of stallion spermatozoa stored during five days (day 1 D1 to day 5 D5) at 5°C FE fresh extended sperm, CC sperm processed through colloidal centrifugation.

	D1		D3		D5	
	FE	CC	FE	CC	FE	CC
Live (A-PI-)%	71.8±15.7^a^	69.5±18.2^a^	47.9±10.6^b^	53.6±12.6^b^	40.0±15.4^b^	48.9±10.2^b^
A+%	6.1±4.3	14.2±15.7	8.5±10.0	8.5±10.0	15.8±15.8	10.3±9.0
A+PI+%	1.0±1.5^a^	0.8±1.1^a^	2.2±2.0	2.8±2.3	2.9±3.9^b^	2.9±3.3^b^
A-PI+%	20.9±14.8^a^	15.6±11.3^a^	41.4±9.2^b^	31.2±15.9^b^	41.2±12.9^b^	37.9±12.8^b^

Live % percentage of live sperm, A+, annexin positive sperm, spermatozoa depicting translocation of PS, A+/PI dead spermatozoa depicting PS translocation, A-PI+ necrotic spermatozoa Within a row values with different superscript differ statistically a-b p<0.01. (Means ± SD) Results are derived from 28 identical experiments (7 stallions, 4 ejaculates per stallion).

### Mitochondrial membrane potential and percentage of Lipid peroxidation (LPO)

There were no changes on the mitochondrial membrane potential related either to the storage period or the sperm processing (table 6). In a similar way LPO was not affected either by the period of storage or the sperm processing.

**Table 6:** Mitochondrial membrane potential (Δϕm) and lipid peroxidation (LPO) of stallion spermatozoa stored during five days (day 1 D1 to day 5 D5) at 5°C FE fresh extended sperm, CC sperm processed through colloidal centrifugation.

	D1		D3		D5	
	FE	CC	FE	CC	FE	CC
High %	4.4±8.8	8.3±12.2	4.4±7.4	5.6±6.2	2.2±4.4	6.2±5.8
High and low%	27.3±16.3	23.2±23.6	23.6±17.3	30.4±22.2	31.4±13.5	10.9±16.8
Low%	68.1±22.5	67.8±33.3	71.9±22.5	63.9±26.9	62.2±17.7	82.9±18.4
LPO%	2.0± 2.4	3.4±5.1	5.0± 5.8	4.1±4.2	3.1±3.1	1.8±1.6

(Means ± SD) Results are derived from 28 identical experiments (7 stallions, 4 ejaculates per stallion). High spermatozoa depicting high Δϕm, High and Low spermatozoa depicting simultaneously mitochondria with low and high Δϕm, Low spermatozoa with low Δϕm, LPO spermatozoa showing peroxidation of the lipids of their membranes.

### LC3B processing to measure autophagy

**Figure 1:**
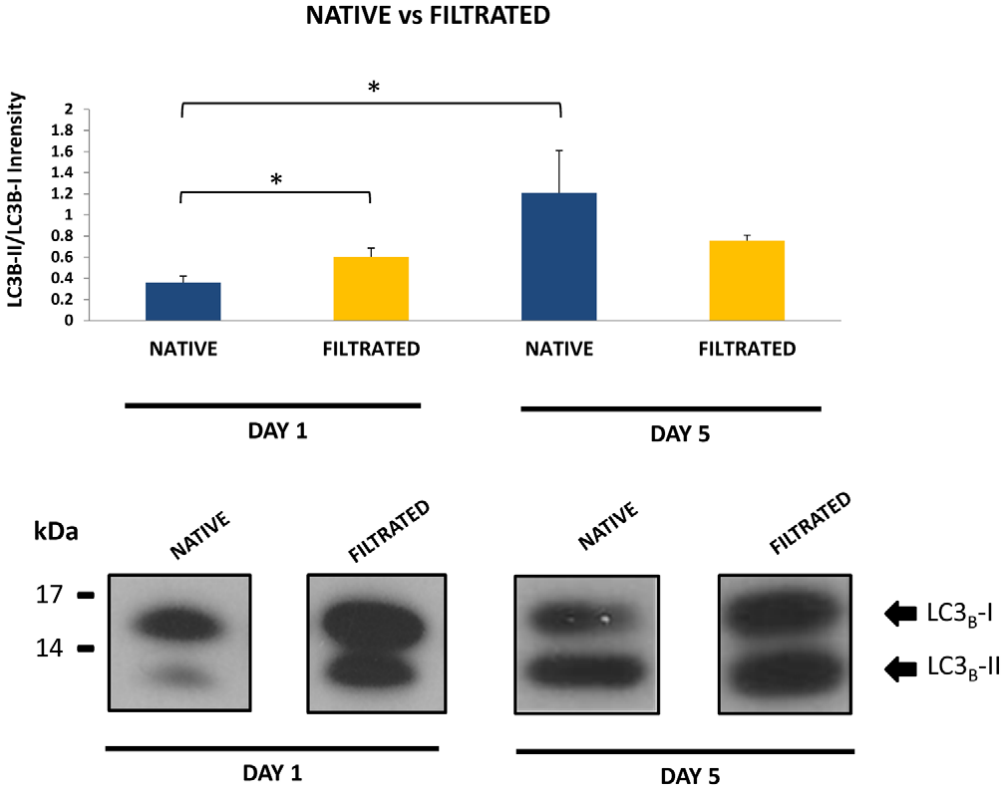
Changes in LC3B processing in stallion spermatozoa stored under refrigeration (5°C) for five days, after single layer centrifugation (Filtrated) or unprocessed (native sperm). In native sperm storage induced a significant increase in LC_3_B processing at day 5 indicating that autophagy was activated during the period of storage. On the other hand, filtration of sperm selected a subpopulation of spermatozoa in which autophagy was already activated at the beginning of the period of storage and did not change over the time. Results are representative of 28 identical experiments (seven stallions, four ejaculates per stallion) * p<0.01.

Measuring processing of endogenous LC3B by western blot is the most common approach to measure autophagy in cells [14]. In native (non filtrated, fresh extended sperm) storage induced a significant increase in LC3B processing at day 5 indicating that autophagy was activated during the period of storage. On the other hand, processed (CC) sperm selected a subpopulation of spermatozoa in which autophagy was already activated at the beginning of the period of storage (figure 1), and did not change over the time.

### Identification and subcellular distribution of TNF, TNFR1 and TNFR2 in stallion spermatozoa

**Figure 2:**
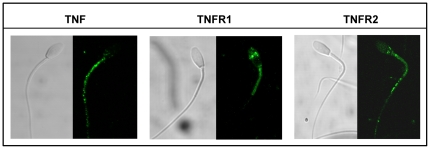
Subcellular distribution of TNF alpha and TNR receptors R1 and R2 in stallion spermatozoa. Their subcellular distribution in fixed and permeabilized stallion spermatozoa was assessed by immunocytochemistry with specific antibodies as described in material and methods. TNF was localized in the mid piece and rest of the tail, TNFR1, was present in the acrosomal region and mid piece, while TNFR2 was present in the post acrosomal region, mid piece and rest of the tail. All images were obtained with a Bio Rad MRC1024 confocal microscope. Magnification, 60x.

The subcellular distribution of TNF, TNFR1 and TNFR2 was investigated in fixed and permeabilized stallion sperm using specific monoclonal antibodies. TNF and both receptors /TNFR1 and TNFR2 were present in stallion spermatozoa (figure 2). TNF was localized in the mid piece and rest of the tail, TNFR1, was present in the acrosomal region and mid piece, while TNFR2 was present in the post acrosomal region mid piece and rest of the tail.

### Correlations among apoptotic markers and the sperm analysis

Positive as well as negative correlations were found among sperm quality and different apoptotic markers. The percentage of motile sperm was positively correlated with the percentage of intact sperm (YoPro-/Eth-) (r = 0.602, p<0.01), live sperm (SYBR-14+/PI-) (r = 0.549, p<0.01) and non apoptotic sperm (A-/PI-) (r = 0.713 p<0.01). Necrotic and apoptotic markers negatively correlated with motility; (YoPro+/Eth+) (r = −0.445, p<0.01), A+/PI+ (r = −0.280 p<0.01), YoPro+/Eth- (r = 0.291 p<0.01) However low caspase 3 and 7 activity positively correlated with motility (r = 0.314 p<0.01). High caspase 3 and 7 activity was correlated with apoptotic sperm (YoPro+, r = 0.312 p<0.01; A+ r = 0.287 p<0.01) and negatively correlated with necrotic sperm (Eth+ r = −0.362 p<0.01) and LPO (r = −0.456 p<0.01). Low caspase activity correlated positively with annexin negative sperm (r = 0.345 p<0.01).

## Discussion

In the present study spermatozoa were investigated for the presence of LC3B in order to determine if the molecular machinery related to autophagy was present; for the first time we found that both LC3B-I and LC3B-II were present in ejaculated stallion spermatozoa. In somatic cells the primary role of autophagy is to protect cells under stress conditions such as starvation. Thus, can be considered that a major function of autophagy is lifespan extension [34], [35] and consequently a strategy for survival in mammalian cells. Both situations -stress and extension of the lifespan of the spermatozoa- are hallmarks of sperm conservation. This is to the authors' knowledge the first report indicating that the mammalian spermatozoa have the molecular machinery necessary for autophagy. It is true that the presence of LC3B may simply represent a remnant from spermatogenesis, in a similar way as has been proposed in the theory of abortive apoptosis[36], for apoptotic markers present in mature spermatozoa. However, autophagy appears to be differentially activated in the spermatozoa, with a subpopulation of sperm that have higher relation of LC3B-II/LC3B-I (Figure 1) on day 1. This subpopulation is also characterized by higher percentage of intact sperm on day 1 but not in day 5, however on day 5 both subpopulations of spermatozoa have similar relation of LC3B-II/LC3B-I intensity. These findings may indicate that an “autophagy like” phenomena may have in sperm distinct functions as has been proposed for somatic cells[37]. On one side this autophagy-like mechanism can be related to sperm survival since the spermatozoa selected through gradient centrifugation had higher percentages of live and intact sperm and also had higher levels of processed LC3B. In the unselected subpopulation there was an increase of processed LC3B after 5 days of storage. This may represent a response of the spermatozoa to nutrient deprivation along the storage period, but taking in account that in the selected population there was not a change in LC3 processing another role for autophagy in sperm can not be completely ruled out. Although autophagy may also lead to programmed cell death in somatic cells [37], [38], [39] it is believed that authophagy is mainly a strategy for survival and the fact that the subpopulation of more viable spermatozoa at the same time had more processed LC3B-II suggest that this is also true for spermatozoa.

Apoptosis is a major cause of sperm damage during cryopreservation in the stallion spermatozoa [17], [29], [40], [41], and in the present study we investigated whether apoptosis also plays a role in sperm aging during cooled storage. Although apoptosis in mature spermatozoa has been under debate due to the fact that are highly differentiated terminal cells, recently new evidences suggest that apoptosis in mature sperm, has a role in the removal of these cells from the male and female reproductive tracts once viability has been lost [42]. Most of the ejaculated spermatozoa die in the female reproductive tract after insemination. Active caspases 3 and 7 were present in fresh sperm, indicating that upon ejaculation an important percentage of spermatozoa are already activated to die through apoptosis. This finding confirms previous reports from our laboratory [16]. Caspase 3 is considered the most important effector caspase, with its activation marking a “point of no return” in apoptosis [43]. In fact, lose of sperm quality seems to be an apoptotic phenomenon [44], [45]; this is further demonstrated in our study by the fact that high caspase 3 and 7 activity was correlated with apoptotic sperm (YoPro + and A+), but at the same time negatively correlated with necrotic sperm (ethidium+). Another fact is that there were no changes in the percentage of live sperm thorough the storage period, while was evidenced a significant increase in the percentage of spermatozoa with increased membrane permeability. Increased membrane permeability is a sign of early apoptosis both in somatic cells [46] and spermatozoa [16], [47]. This increase in membrane permeability is related to the fact that apoptotic cells release ‘find-me’ signals at the earliest stages of death to recruit phagocytes. The nucleotides ATP and UTP represent one class of “find-me” signals that are released by mechanisms that imply increases in membrane permeability [46], [48], using the same channels to be released from the cell as YoPro-1 uses to enter the cell. This fact has also implications for other areas of sperm reproductive technology; since more classical approaches to measure sperm quality such as the combination SYBR-14/PI does not allow detect spermatozoa that have already initiated a way to cell death. Assays capable to detect apoptotic spermatozoa can identify defective spermatozoa at an early stage of damage and thus can be better assays of sperm quality [26], [49]. The use of YoPro-1 is considered a simple, quick, inexpensive and repeatable method to detect apoptosis in somatic cells [50] and spermatozoa [51] and is routinely used in our laboratory to detect early sperm damage [24], [25], [29], [52]. Our findings support the superior value of this technique for sperm analysis confirming previous reports [51], [53]. Another marker of apoptosis that also revealed differences along the incubation period was the monitoring of PS translocation. During more advanced stages of apoptosis dying cells display in the outer membrane PS that is recognized by phagocytes to remove these cells without an inflammatory response, being an “eat-me” signal [18], [54], [55]. At the end of the storage period there was an increase in sperm depicting PS translocation further supporting the existence of apoptotic sperm death during cooled storage.

Sperm motility is routinely used as the main indicator of sperm quality under clinical settings [56], [57]. In our study we found that lose of motility correlated with the percentage of necrotic and also apoptotic sperm; this fact supports the assumption that stallion spermatozoa dye both through necrotic and apoptotic mechanisms. Interestingly lose of motility correlated better with intact spermatozoa than with live sperm, adding value to assays able to detect apoptotic spermatozoa.

During cryopreservation stallion spermatozoa experiences apoptosis involving the intrinsic pathway [16], however in our experiment there were not changes in mitochondrial membrane potential, or changes in LPO, suggesting either that the intrinsic pathway does not play a role in sperm death during cooled storage or that this pathway is already activated at ejaculation. The extrinsic pathway of apoptosis is activated by tumor necrosis factor family of receptors; activation of these receptors results in activation of caspase 8 [54]. The existence of the extrinsic- receptor mediated pathway of apoptosis in spermatozoa is still controversial, but caspase 8 has been identified in human sperm [58], [59] and caspase 8 has been detected previously by flow cytometry in our laboratory, and increased after exposition of stallion sperm to anisosmotic conditions (unpublished). Thus both pathways of apoptosis may be present in stallion spermatozoa. Furthermore we investigated if receptor mediated form of programmed cell death could be present in stallion spermatozoa investigating by immunohistochemistry the presence of TNR receptors and ligand. Both receptors TNR1 and TNFR2 and the TNF ligand were present in stallion spermatozoa indicating that TNF mediated cell death can be present. Activation of TNF receptors can lead in somatic cell to both caspase dependent (apoptosis) or caspase independent (necroptosis) programmed cell death in presence of non-especific caspase inhibitors [60]; this fact offer an explanation to the failure of experiments attempting to reduce cryodamage in sperm using caspase inhibitors [61].

To summarize, major findings of our study are: (i) discovery that LC3B is differentially activated during cooled storage of stallion spermatozoa, (ii) that during cooled storage stallion spermatozoa experiences apoptotic damage (iii) demonstrates the major value of the YoPro-1 assay for the early detection of defective spermatozoa (iv) opens clues to disclose, whether other forms of programmed cell death (necroptosis) occur in the spermatozoa, and more importantly (v) may open new strategies of sperm conservation based in the modulation of cellular pathways leading to sperm death, or enhancing mechanisms of sperm survival.
